# Progress in the Treatment of Refractory Myasthenia Gravis

**DOI:** 10.31083/RN47260

**Published:** 2026-02-26

**Authors:** Dan Liu, Jialing Mao, Jing Song, Manxia Wang

**Affiliations:** ^1^Department of Neurology, Lanzhou University Second Hospital, 730000 Lanzhou, Gansu, China

**Keywords:** refractory myasthenia gravis, targeted therapy, complement inhibitors, CAR-T cell therapy

## Abstract

Myasthenia gravis (MG) is an autoantibody-mediated, cellular immune-dependent and complement system-involved autoimmune disorder characterized by acquired neuromuscular junction transmission dysfunction driven by genetic and environmental factors. Approximately 10% therapies such as cholinesterase inhibitors, glucocorticoids, and immunosuppressants, resulting in the development of refractory MG (RMG). The current emergence of new therapeutic strategies such as targeted biologics (e.g., complement inhibitors, Fc receptor (FcRn) antagonists, etc.), B-cell depletion therapy, and Chimeric Antigen Receptor (CAR)-T cell therapy contribute to the significant improvement in the clinical management of RMG. Accordingly, the present study systematically reviewed the treatment progress of RMG, aiming to provide evidence-based individualized treatment decision-making clinically, alleviate patients' pain, and explore future research directions.

## 1. Introduction

Myasthenia gravis (MG) is an autoantibody-mediated autoimmune disease involving the skeletal muscles of the entire body, characterized by acquired neuromuscular junction (NMJ) transmission dysfunction [1]. The clinical hallmark is muscle weakness that worsens with activity and improves with rest. Despite standard therapies, approximately 10%–20% of MG patients do not achieve adequate disease control, falling under the category of refractory MG (RMG). A universal consensus definition is lacking; however, RMG is commonly defined in clinical studies as the failure to achieve minimal manifestation status (MMS) or remission despite adequate trials of corticosteroids and at least two immunosuppressive agents, or the inability to reduce immunosuppression without relapse [2]. This operational definition underscores the persistent disease activity and significant burden faced by this patient subgroup, for whom conventional strategies have been exhausted.

**Fig. 1.**
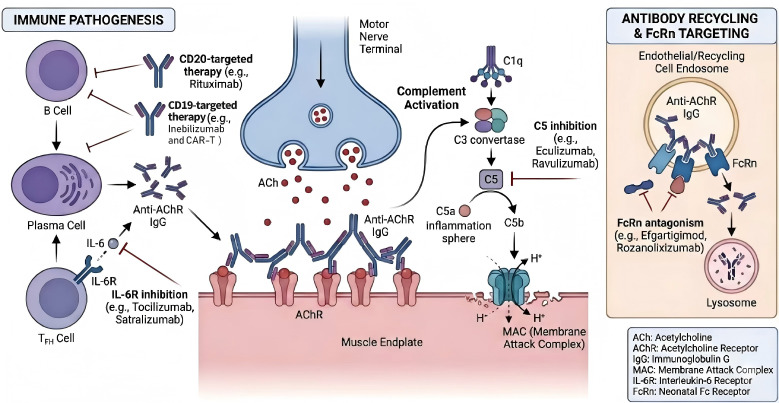
**Immunopathogenesis of myasthenia gravis and sites of action for targeted therapies**. (Adapted from Cavalcante *et al*., 2024 [5], with permission). A horizontal line and a vertical line represent drug action targets, and a unidirectional arrow indicates that the mechanism of action proceeds to the next step. The figure was created using Adobe Illustrator 2023 (Adobe, San Jose, CA, USA).

The pathogenesis of MG involves a complex interplay of humoral and cellular immunity. Pathogenic immunoglobulin G (IgG) autoantibodies are produced by autoreactive B cells and plasma cells, targeting postsynaptic NMJ antigens such as the acetylcholine receptor (AChR), muscle-specific kinase (MuSK), and lipoprotein receptor-related protein 4 (LRP4) [3, 4]. These antibodies impair synaptic transmission through three primary mechanisms: complement activation leading to membrane attack complex (MAC) formation and destruction of the postsynaptic membrane; antigenic modulation and internalization of AChR; and functional blockade of receptor binding. Furthermore, the neonatal Fc receptor (FcRn) plays a crucial role in extending the half-life of pathogenic IgG. Pro-inflammatory cytokines, such as interleukin-6 (IL‑6), contribute to B-cell activation and plasma cell survival. These antibodies may affect the aggregation of AchR on the postsynaptic membrane in a direct or indirect manner, leading to disorders in the transmission of neuromuscular signals, and the development of pathological conditions ultimately. This multifaceted immunopathology provides the rationale for targeted therapies aimed at specific components of this pathway (Fig. 1) [5].

Given the pathogenesis of MG, MG is primarily treated by reducing antibody production, clearing antibodies, and mitigating antibody effects. It is primarily treated by thymectomy, therapeutic plasma exchange (TPE), drug therapy, etc. Among these, drug therapy mainly includes cholinesterase inhibitors, glucocorticoids, immunosuppressants, intravenous immunoglobulin (IVIG), and targeted monoclonal antibodies. However, given that some patients are unable to tolerate adverse drug reactions, they may not be able to use a conventional treatment drug in sufficient doses or still cannot achieve the treatment goals of disease remission (CSR or PR) or MMS even if sufficient doses are used. In severe cases, their condition may recur and worsen, with a heavier medical burden and higher hospitalization and mortality rates [6]. Therefore, it is urgent to break through the limitations of traditional treatment for these RMG patients.

## 2. Tacrolimus

Although this review emphasizes novel biologics, tacrolimus-a calcineurin inhibitor, warrants mention due to its continued role in specific RMG contexts. Its inclusion is justified by its cost-effectiveness, oral administration, and utility in resource-limited settings or as a bridging therapy. Tacrolimus, also known as FK506, is a neurocalmodulin inhibitor that exerts immunosuppressive effects by inhibiting the neurocalmodulin phosphatase pathway, reducing the proliferation of activated T cells, and promoting nerve fiber regeneration [7]. Tacrolimus can also increase muscle strength by enhancing the function of the RyR [8]. A foreign study has retrospectively documented that tacrolimus monotherapy can alleviate MG symptoms rapidly with fewer adverse reactions, outperforming traditional immunosuppressants [9]. Moreover, tacrolimus can significantly improve the clinical symptoms of RMG patients and reduce corticosteroid therapy dosage [10, 11], which can also increase the proportion and quantity of regulatory T cells to reduce the autoimmune response of MG patients [12]. Strictly speaking, tacrolimus is a traditional therapy that has currently been one of the major choices for clinical treatment of RMG given its positive efficacy in RMG patients and lower cost compared to other biologically targeted drugs. It represents a conventional yet sometimes necessary option before or alongside access to more targeted biological agents.

## 3. Azathioprine

Azathioprine, a purine analogue immunosuppressant, remains one of the most widely used corticosteroid-sparing agents in the long-term management of MG, including in some cases before the designation of refractoriness [13]. Its mechanism involves inhibiting DNA and RNA synthesis, thereby reducing the proliferation of lymphocytes. While its onset of action is slow (often 3–6 months), it is effective in maintaining remission and reducing cumulative glucocorticoid exposure. Its use requires careful monitoring for myelosuppression and hepatotoxicity [14]. Although not typically a primary agent for established refractory MG, its role in the therapeutic sequence leading up to refractoriness and its presence in treatment guidelines warrant its mention here.

## 4. Intravenous Immunoglobulin (IVIG)

Immunoglobulin is rich in γ-globulin obtained from highly purified human plasma. IVIG can disrupt the binding function of antigens and antibodies to interfere with immune responses, which has been used to treat various autoimmune diseases [15, 16]. For RMG patients, IVIG can realize a rapid recovery from severe acute exacerbations [17, 18] and a reduction in the dosage of other immunosuppressive drugs. While subcutaneous immunoglobulin (SCIG), an alternative to IVIG, has been documented to possess advantages of fewer side effects, flexible infusion time, and patient independence [19]. As proven by an open phase II clinical trial, SCIG exhibited excellt safety and tolerability within 12 weeks, which was more effective than IVIG in maintaining stable disease [20]. Therefore, therapeutic options for RMG are expected to be expanded based on its safety and efficacy.

## 5. B-Cell Targeted Drugs

Rituximab is a human-mouse chimeric monoclonal antibody of the IgG1κ class, with the ability to target CD20 on the surface of B cells. On this basis, it may trigger complement-mediated cytotoxicity, resulting in the depletion of CD20-positive B cells in the human body. In a prior meta-analysis on the treatment of RMG using Rituximab, the overall clinical symptom improvement rate of Rituximab was 77% and 73% for the treatment of serum anti-AChR and anti-MuSK-positive or anti-AChR and anti-MuSK-negative RMG, respectively, with the daily corticosteroid therapy reduced by 21.70 (15.50, 42.46) mg/d [21], indicating its significant efficacy and its role in lowering corticosteroid therapy. Meanwhile, a single low-dose of Rituximab for RMG can effectively alleviate the clinical symptoms of such patients and reduce corticosteroid therapy [22], and its therapeutic efficacy was independent of the duration of the disease [23]. However, compared to RMG patients with disease duration >1 year and for those without response to multiple immunosuppressive agents, new-onset generalized MG (gMG) patients required less time to achieve symptom relief after Rituximab injection, accompanied by fewer rescue incidents during treatment and less additional immunotherapy [24]. At this stage, these findings require confirmation through further research.

Furthermore, Ofatumumab is a second-generation fully human IgG1 anti-CD20 monoclonal antibody that can bind tightly to two unique epitopes of CD20 and slowly sheds, inducing complement-dependent cytotoxicity and antibody-dependent cell-mediated cytotoxicity in CD20-expressing B cells [25]. It has been applied to treat adult relapsing multiple sclerosis [26]. In a case study abroad, an RMG patient with poor response to Rituximab continued to experience symptom relief following Ofatumumab treatment [27]. A recent case report in China also demonstrated the effect of Ofatumumab administration in rapidly clearing B cells, quickly reducing corticosteroid therapy and significantly improving clinical symptoms in an anti-AchR-positive RMG patient. Meanwhile, there were no adverse reactions and disease recurrence during the 8-month follow-up [28]. However, in current research, some patients still experience recurrence after B cell reconstruction, highlighting the further requirement for exploring maintenance treatment strategies. Moreover, we may know little about the potential presence of differences in their efficacy given the absence of antibody subtype (Titin, LRP4) stratification in existing clinical trials. Further research is still needed to confirm the clinical efficacy of Ofatumumab.

Besides, Inebilizumab represents a significant advancement in the therapeutic landscape for refractory generalized MG. As a humanized, glycoengineered monoclonal antibody, it targets the CD19 antigen, offering a distinct and potentially more comprehensive mechanism of B-cell depletion compared to earlier therapies. Its recent approval, based on robust phase 3 trial data, has positioned it as an important new option for patients with anti-AChR antibody-positive MG who have not adequately responded to standard treatments [29]. The pathophysiology of MG is driven by B cells that produce pathogenic autoantibodies, primarily against the AChR at the neuromuscular junction. While the anti-CD20 antibody rituximab has been used off-label with success, it primarily depletes mature B cells and some plasmablasts, but spares early B-cell precursors and a significant proportion of antibody-secreting plasmablasts and plasma cells [30]. Inebilizumab’s key differentiating factor is its target CD19. This antigen is expressed more broadly on the B cell lineage, from early pro-B cells through mature B cells, and crucially, on plasmablasts and a subset of plasma cells. By binding to CD19, inebilizumab induces antibody-dependent cellular cytotoxicity and complement-dependent cytotoxicity, leading to the depletion of a wider spectrum of B lineage cells. This broader depletion strategy aims to more effectively reduce the source of pathogenic autoantibodies, potentially leading to faster and more sustained clinical improvement [29].

So far, several on-going studies are carried out to study the therapeutic effects of other drugs targeting B cells (e.g., Belimumab, Zanubrutinib, Abatacept, Upadacitinib, Obinutuzumab, etc.) on MG. These drugs may be a new choice for RMG patients in the future considering their effects of directly or indirectly clearing B cells, inhibiting B cell activation and proliferation, and improving the immune microenvironment.

## 6. Inhibitor of Complement C5

Eculizumab is a humanized anti-IgG2/4 κ monoclonal antibody targeting complement C5. It works to exert therapeutic effects by reducing damage to the NMJ and loss of AChR and exerting therapeutic effects resulting from inhibiting the conversion of C5 to C5a and C5b, as well as blocking complement activation and the formation of membrane attack complex (MAC). Eculizumab is currently the only drug validated through a phase III clinical trial for RMG treatment [31]. Moreover, it has been approved for treating AchR-positive refractory gMG, with confirmed safety and efficacy [29, 30, 31]. A study abroad compared the changes in the Quality of Life in Neurological Disorders (Neuro-QOL) scores, MG Activities of Daily Living Scale, Quantitative MG Score, and MG Quality of Life 15-item Scale scores of AchR-positive refractory gMG patients with Eculizumab and placebo interventions. Consequently, patients treated with Eculizumab had significantly better Neuro-QOL fatigue scores than those treated with placebo, indicating its effect in improving the quality of life and fatigue symptoms of RMG patients [32]. Furthermore, switching to Eculizumab can achieve the treatment goals of MMS for most patients without significant adverse reactions when managing RMG patients with persistent symptoms without improvement in the context of Rituximab treatment history [33]. In addition, there has been a report on two AChR-positive refractory and MG Foundation of America (MGFA) V-grade gMG cases who had no response to IVIG, TPE, prednisone, and Rituximab. However, the two patients showed a strong response to Eculizumab, coupled with obvious improvement in MGFA classification, MG-ADL scale, and MG composite scale indicators [34]. Nevertheless, this therapy has not been widely applied at present due to the limited clinical sample size of existing research and the high cost of this therapeutic agent.

In addition, Zilucoplan and Ravulizumab, two C5 complement inhibitors, have achieved positive outcomes in clinical trials for MG treatment. Specifically, Zilucoplan is a synthetic macrocyclic peptide [35] that can block the cleavage of complement C5 into C5a and C5b, inhibit MAC formation, and eventually inactivate the terminal pathways of the complement system, as well as reduce inflammation and tissue damage [36]. It has achieved positive results in treating gMG, with good safety and efficacy [37, 38] and long-term sustained curative effects [39]. Currently, Zilucoplan has been approved for managing gMG in anti-AChR-positive adult patients [40]. Furthermore, Ravulizumab is a long-acting humanized IgG2/4κ monoclonal antibody against complement C5, revealing stronger pharmacokinetic properties compared to Eculizumab [41]. Existing phase III clinical trials have revealed its long-term efficacy and good safety in the treatment of patients with AchR-positive gMG [42]. In a study by Japanese scholars, among 36 RMG patients with prior treatment history using Eculizumab, functional impairment was observed in 6 of the 13 patients with Eculizumab withdrawal, while the other 15 patients who switched from Eculizumab to Ravulizumab exhibited good tolerability and efficacy and were more inclined towards Ravulizumab treatment [43]. Therefore, Zilucoplan and Ravulizumab, given their similar mechanism to Eculizumab, are expected to be better choices for RMG treatment, with further clinical exploration required to confirm their efficacy.

## 7. Fc Receptor (FcRn) Antagonist

Efgartigimod is a modified human IgG1-derived Fc fragment, enabling a binding to the human FcRn to lower pathogenic IgG antibodies and block IgG circulation, thereby achieving endogenous clearance of IgG. It has been approved for and has achieved good therapeutic results for the treatment of AchR-positive adult systemic MG in China. Given its therapeutic mechanism, it is expected to treat various categories of RMG independent of antibody types. Moreover, another report mentioned significant improvement and control [44] in the condition in a refractory gMG patient with multiple immunotherapy failures receiving the administration of Efgartigimod, coupled with a rapid correction in myasthenia crisis [45, 46, 47]. A foreign study also investigated a patient with serum triple-negative refractory gMG for 28 years, associated with repeated involvement of the medulla oblongata and systemic weakness, multiple treatment failures and MG crisis. The application of Efgartigimod treatment led to significant improvement in clinical symptoms (MGFA: IIIb to IIb; MG-ADL rating: 11 to 0; MG-QOL15 rating: 30 to 0; QMG score: 28 to 6) in this patient during the five cycles of treatment [48]. In addition, the efficacy and safety of Efgartigimod in treating RMG has been supported by a retrospective analysis in the UK [49].

Besides, humanized FcRn antagonists [e.g., Rozanolixizumab (UCB-7665), Nipocalimab, Batoclimab, etc.] can increase IgG clearance without affecting IgG production or serum levels of IgA, IgD, IgE, and IgM and reduce the risk of infection. The key phase III clinical trials for Rozanolixizumab and Nipocalimab also demonstrated their significant clinical benefits and good safety in both AchR-positive and MuSK-positive gMG [50, 51]. Rozanolixizumab was approved for marketing in China in March 2025 for treating adult patients with systemic MG, while Nipocalimab was approved by the US FDA in April 2025 for patients with systemic MG. Additionally, another Phase 2a open-label extension study of Batoclimab also verified its clinical benefits for gMG [52]. Collectively, these therapeutic agents may exhibit more advantages and application prospects in future RMG treatment.

## 8. Antagonists Targeting Cytokines and Cytokine Receptors

As a recombinant humanized IgG1 monoclonal antibody, Tocilizumab targets the interleukin (IL)-6 receptor. It is currently used to treat rheumatoid arthritis, systemic juvenile idiopathic arthritis, giant cell arteritis, cytokine release syndrome, etc. However, two RMG patients who were unresponsive to Rituximab experienced improved clinical symptoms after Tocilizumab treatment [53]. Another cohort study prospectively reported that for refractory anti-AChR-positive gMG patients, Tocilizumab can significantly improve the MG-ADL score and reduce corticosteroid therapy without serious safety issues [54], indicating the therapeutic potential of Tocilizumab for RMG. In addition, other monoclonal antibodies within the same family, such as Brodalumab, Ixekizumab, and Secukinumab targeting IL-17 and IL-17A, or Ustekinumab targeting IL-12 and IL-23, may also have the potential to treat MG. At this stage, further investigation is required to confirm these findings.

Satralizumab is a humanized monoclonal antibody targeting the interleukin-6 receptor (IL-6R). By inhibiting the IL-6 pathway, it modulates B-cell differentiation and plasma cell survival, which are implicated in MG pathogenesis. While its primary approval is for neuromyelitis optica spectrum disorder, preliminary evidence and its mechanism support its potential investigation in refractory MG, particularly in patients with an IL-6-driven pathophysiology [55]. Clinical trials are warranted to establish its efficacy and safety profile specifically in the MG population.

## 9. Innovative Therapy: Chimeric Antigen Receptor (CAR)-T-Cell Therapy

For MG patients, autoantibody-producing plasma cells (PCs) constitute a key cellular component of MG pathophysiology, and B-cell mature antigen (BCMA) is usually significantly expressed in plasmablasts and PCs, underscoring its significance as a promising target antigen of new therapies for serum autoantibody-positive MG. Chimeric antigen receptor-T (CAR-T) cell therapy is a novel immunotherapy modifying patients’ T cells via genetic engineering to express specific chimeric antigen receptors, thereby specifically recognizing and killing target antigens harbored by target cells. Therefore, it may be applicable to apply CAR-T cells that recognize BCMA-positive B cells for the treatment of RMG, given the pathogenesis and pathophysiological processes of MG. In a foreign clinical trial on MG patients, CAR-T cells were modified using BCMA targets and mRNA technology in 14 recruited patients. Patients undergoing CAR-T cell therapy showed significantly reduced MG-ADL and QMG scores, obviously lowered antibody titers or even negative conversion, and no relevant toxic side effects [56]. Furthermore, CAR-T cell therapy targeting BCMA achieved good safety and clinical improvement for over 18 months in two patients with recurrent MG and RMG [57]. Moreover, for muscle-specific tyrosine kinase (MuSK) MG, MuSK chimeric autoantibody receptor T cells were designed specifically by foreign researchers, which covered the complete extracellular domain of MuSK and the ζ signaling domain of CD137-CD3. Experimental models have documented that it can specifically kill B cells expressing anti-MuSK B cell receptors and reduce anti-MuSK IgG without reducing total B cells and IgG. In addition, its safety has been verified through various methods, and this method has now entered the clinical trial application stage [58]. Altogether, CAR-T cell therapy can significantly improve the clinical symptoms of RMG patients, which is expected to offer fresh therapeutic options for patients with RMG in the future.

## 10. Practical Management Considerations and Patient Subgroups

AChR-Positive RMG: First-line biologic options include complement C5 inhibitors (high efficacy, require monitoring/vaccination) or FcRn antagonists (broad applicability, rapid onset, cyclical dosing) [59]. Rituximab is a valid alternative, particularly with comorbid autoimmune conditions. MuSK-Positive RMG: Rituximab is the best-supported first-line biologic [60]. FcRn antagonists also demonstrate clear efficacy. Seronegative RMG: FcRn antagonists are a rational first choice [61]. B-cell-depleting therapies may be considered based on clinical phenotype [62]. Dosage, administration, and key monitoring points for major agents are summarized in Table 1.

**Table 1.** **Comparison of novel biologic therapies for refractory myasthenia gravis**.

Drug class	Mechanism	Key indications	Administration & Typical dosing	Common adverse events	Key monitoring
C5 Inhibitor (Eculizumab)	Blocks terminal complement	AChR+gMG	IV, every 2 weeks	Headache, URTI, meningococcal risk	Vaccination, infection signs
C5 Inhibitor (Zilucoplan)	Blocks terminal complement	AChR+gMG	SC, daily self-injection	Injection site reaction, URTI	Vaccination, infection signs
C5 Inhibitor (Ravulizumab)	Blocks terminal complement	AChR+gMG	IV, weight-based dosing every 8 weeks	Headache, URTI, meningococcal risk	Vaccination, infection signs
FcRn Antagonist (Efgartigimod)	Accelerates IgG degradation	AChR+MuSK+SNMG	IV or SC, cyclical	Headache, diarrhea	IgG levels
FcRn Antagonist (Rozanolixizumab)	Accelerates IgG degradation	AChR+MuSK+SNMG	SC, weight-based cyclical dosing	Headache, diarrhea, injection site reactions	IgG levels
Anti-CD20 (Rituximab)	Depletes CD20+ B cells	MuSK+AChR+	IV, induction + maintenance cycles	Infusion reactions, infections, PML risk	CD19+ B‑cell count, infection signs
Anti-CD19 (Inebilizumab)	Depletes broad B-lineage cells	AChR+MuSK+	IV, induction + maintenance	Infusion reactions, infections	CD19+ B‑cell count, infection signs
Anti-IL-6R (Tocilizumab/Satralizumab)	Blocks IL-6 receptor	Refractory (AChR+ gMG)	IV/SC for Tocilizumab; SC for Satralizumab	Infections, increased liver enzymes, cytopenias	Infection signs, liver function tests, complete blood count
CAR-T (BCMA)	Depletes BCMA+ plasma cells/B cells	Highly refractory AChR+	Single IV infusion	CRS, ICANS, cytopenias, infection	Intensive monitoring for CRS/ICANS, blood counts, immunoglobulins

gMG, generalized Myasthenia Gravis; SNMG, Seronegative MG; IV, Intravenous; SC, Subcutaneous; URTI, Upper Respiratory Tract Infection; PML, Progressive Multifocal Leukoencephalopathy; CRS, Cytokine Release Syndrome; ICANS, Immune Effector Cell-Associated Neurotoxicity Syndrome; PML, Progressive Multifocal Leukoencephalopathy; MuSK, muscle-specific kinase; AChR, acetylcholine receptor; BCMA, B-cell mature antigen; IL-6R, interleukin-6 receptor; CAR-T, chimeric antigen receptor-T.

## 11. Proposed Treatment Algorithm

**Fig. 2.**
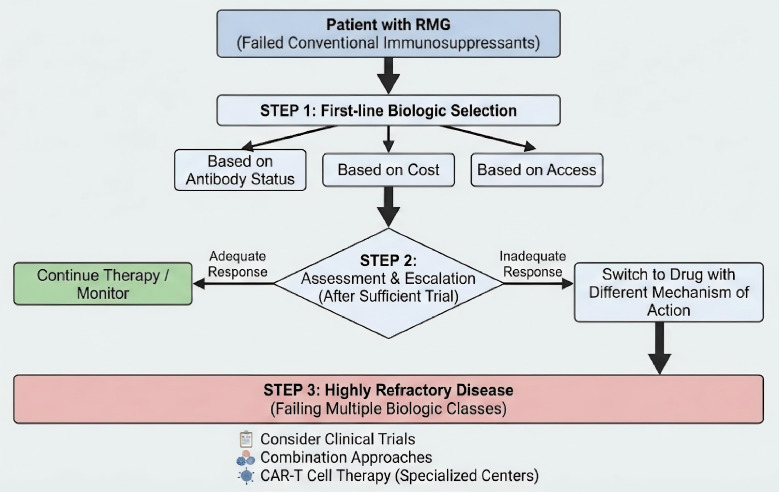
**Proposed treatment algorithm for refractory myasthenia gravis**. Note: This treatment algorithm was created by the authors based on the evidence synthesized in this review. RMG, refractory myasthenia gravis. The figure was created using Adobe Illustrator 2023.

Synthesizing the available evidence, we propose a practical treatment algorithm (Fig. 2) for managing RMG after conventional immunosuppressants have failed. First-line biologic selection is choosing based on antibody status, cost, and access. The second is assessment and escalation, if the response is inadequate after a sufficient trial, switch to a drug with a different mechanism of action. The third is highly refractory disease, for patients failing multiple biologic classes, consider clinical trials, combination approaches, or CAR-T cell therapy in specialized centers.

## 12. Conclusions

In summary, significant progress has been made in RMG treatment. The emergence of bio-based targeted drugs (e.g., Rituximab, Eculizumab, Ofatumumab, and Efgartigimod) and innovative CAR-T cell therapy can overcome the limitations of traditional therapies, rapidly improve patients’ clinical symptoms, and provide assistance to RMG patients. However, there are still many challenging issues, such as the long-term safety and efficacy of these therapeutic agents in RMG patients with different antibody types and serum negativity, and the development of other effective drugs of the same type for RMG patients. Moreover, the follow-up data for most new drugs is less than 5 years, requiring further observational studies with prolonged duration. CAR-T cell therapy exhibits relatively optimistic safety results so far due to the limited number of patients currently. CAR-T cell and its derivative therapies are innovative strategies for MG, which, however, remain at their early exploration stage. In addition, there is an absence of large-scale clinical trial or long-term follow-up data support for specific antibodies, coupled with high cost of CAR-T cell therapy. Its value for widespread application deserves further exploration to confirm its long-term efficacy and economic benefits. Overall, the therapeutic paradigm of RMG is undergoing revolutionary changes. In the future, our emphasis should still be attached to the translational medicine research (e.g., biomarker development and drug resistance mechanism elucidation).
